# Comparative Study of Chemical Stability of Two H_1_ Antihistaminic Drugs, Terfenadine and Its *In Vivo* Metabolite Fexofenadine, Using LC-UV Methods

**DOI:** 10.1155/2019/5790404

**Published:** 2019-04-02

**Authors:** Anna Gumieniczek, Anna Berecka-Rycerz, Rafał Pietraś, Izabela Kozak, Karolina Lejwoda, Paweł Kozyra

**Affiliations:** Department of Medicinal Chemistry, Medical University of Lublin, Jaczewskiego 4, 20-090 Lublin, Poland

## Abstract

A comparative study of chemical stability of terfenadine (TER) and its *in vivo* metabolite fexofenadine (FEX) was performed. Both TER and FEX were subjected to high temperature at different pH and UV/VIS light at different pH and then quantitatively analyzed using new validated LC-UV methods. These methods were used to monitor the degradation processes and to determine the kinetics of degradation for both the compounds. As far as the effects of temperature and pH were concerned, FEX occurred more sensitive to degradation than TER. As far as the effects of UV/VIS light and pH were concerned, the both drugs were similarly sensitive to high doses of light. Using all stress conditions, the processes of degradation of TER and FEX followed the first-order kinetics. The results obtained for these two antihistaminic drugs could be helpful in developing their new derivatives with higher activity and stability at the same time.

## 1. Introduction

**Figure 1:**
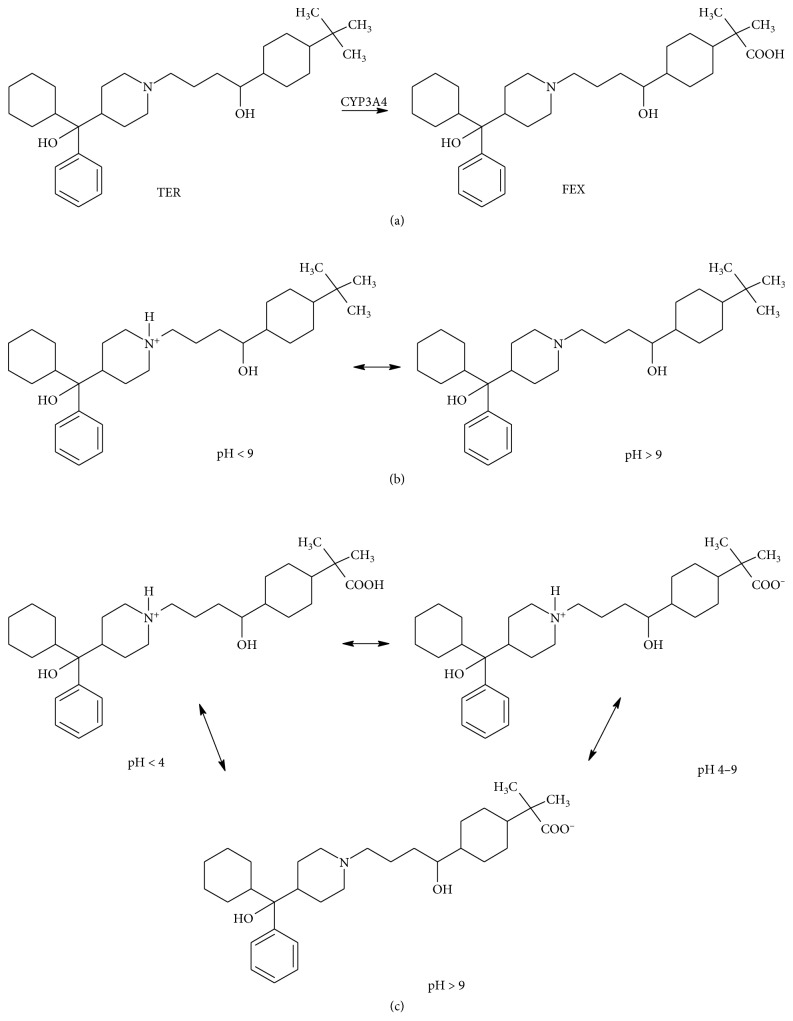
(a) *In vivo* conversion of terfenadine (TER) to fexofenadine (FEX); (b) isocationic and neutral forms of TER; (c) isocationic, neutral, and isoanionic forms of FEX.

All therapeutic substances undergo constant changes when they are influenced by external factors such as temperature, humidity, acidic or alkaline pH, and UV/VIS light. At the same time, all final pharmaceutical products should maintain the appropriate potency and purity throughout the entire period of their availability in the market [1]. Terfenadine (TER) and fexofenadine (FEX) belong to the second generation of H_1_ antihistamines with a significantly lower sedative action compared to the first generation. They both bind selectively peripheral H_1_ receptor and consequently provide amelioration of histamine-induced symptoms [2, 3]. Today, it is known that TER shows a strong affinity for some types of potassium channels, which is associated with cardiac abnormalities. This was the reason for the withdrawal of TER from the pharmaceutical market. FEX is an active metabolite of TER formed *in vivo* by transforming the *tert*-butyl to the carboxylate group under the influence of the enzyme CYP3A4 (Figure 1(a)). On the contrary to TER, FEX provides a sufficient balance between efficacy and safety for the cardiovascular system and is widely used for the treatment of allergy symptoms [4, 5].

The research in the field of antihistaminic activity of different compounds is continuous ongoing study, and some reports concerning novel analogues of TER and FEX are present in the literature. In addition, TER was shown to manifest additive biological effects such as anticholinergic or antiviral activities [6–8]. Based on these results, a series of TER derivatives was prepared in order to increase these activities and to derive new structure-activity relationships [7, 8]. What is more, TER was shown to be effective against carcinogenesis in multiple cancer models and to restore activity of many anticancer agents. Therefore, TER or its chemical derivatives may be a promising approach in some types of cancer [9, 10].

Only few HPLC methods for determination of TER have been reported so far. They were mainly elaborated for different pharmacokinetic measurements [11–14]. However, more HPLC methods were elaborated for determination of FEX in pharmaceuticals, as an individual drug [15–18] or in the presence of other agents like pseudoephedrine [19] or parabens [20]. Some of the methods from the literature were described as stability-indicating procedures capable of determining FEX in the presence of its degradation products [20–24].

As far as chemical stability of TER is concerned, there is no previous report in this area. On the contrary, some data presenting insufficient stability of FEX occurred in the literature [15, 16, 19, 21–24]. Its chemical stability was previously examined in a solid state at 80°C for 1–8 h [21, 22] as well as in solutions, i.e., in 0.1–1 M HCl, 0.1–1 M NaOH, and 3–30% H_2_O_2_ [16, 17, 19, 21–23]. As far as photodegradation of FEX is concerned, the exposure of FEX to the day light and irradiation at 254 nm was carried out [16, 21–24].

In the present experiment, a comparative study concerning stability of TER and FEX was performed, keeping in view their chemical structures, i.e., replacement of *tert*-butyl of TER by the carboxylate group of FEX. Both TER and FEX were subjected to high temperature or UV/VIS light, in solutions over a wide pH range. The applied doses of light were equal or 2–5 times higher than the dose of light used routinely for confirming photostability of new drugs [25]. In addition, new validated LC-UV methods were elaborated and applied for quantitative determinations of TER and FEX in the stressed samples. The obtained levels of degradation as well as kinetic parameters were calculated and used to compare both these drugs.

## 2. Materials and Methods

### 2.1. Materials

Pharmaceutical-grade standards of terfenadine (TER), fexofenadine hydrochloride (FEX), amiodarone hydrochloride, papaverine hydrochloride, and starch purchased from Sigma-Aldrich, St. Louis, MO, USA; acetonitrile, methanol, sodium hexanesulfonate, and triethylamine (TEA) from Merck, Darmstadt, Germany; acetic acid (CH_3_COOH), sodium acetate (CH_3_COONa), hydrochloric acid, sodium chloride (NaCl), sodium tetraborate (Na_2_B_4_O_7_), sodium hydrogen phosphate (NaHPO_4_), sodium hydroxide (NaOH), kalium dihydrogen phosphate (KH_2_PO_4_), and kalium hydroxide (KOH) from POCh, Gliwice, Poland were used. Buffers for LC methods (acetate buffers of pH 3.1 and 4.8) as well as buffers for degradation (acetate buffer of pH 4.0, phosphate buffer of pH 7.0 and borate buffer of pH 10.0) were prepared as described in European Pharmacopoeia [26]. The buffers used for degradation have the same ionic strength of 1 M, which was attained with 4 M NaCl.

### 2.2. Stock Solutions

Stock solutions of TER, FEX, and both i.s. were prepared in methanol at a concentration of 1 mg/ml. These solutions were stored in dark at 4°C, and all were found to be stable for several weeks.

### 2.3. LC-UV Methods

#### 2.3.1. Chromatography and Validation

Chromatography was performed with a model 306 pump with a loop Rheodyne (20 *μ*l) and a model UV170 detector controlled by OMNIC software (all from Gilson Inc., Middleton, WI, USA). The columns were housed in a column heater set at 25°C. The developed methods were validated according to the ICH and FDA guidelines for their specificity, linearity, sensitivity, accuracy, precision, and robustness [27, 28].

#### 2.3.2. Chromatographic Conditions for TER

Assay of TER was carried out on a LiChrospher®C8 column (125 × 4.0 mm, 5 *μ*m) purchased from Merck. The mobile phase consisted of acetonitrile, methanol, and acetate buffer of pH 4.8 (50 : 30 : 20, v/v/v). The flow rate of the mobile phase was 1.0 ml/min. Detection was done at 220 nm, while amiodarone was used as an internal standard (i.s.).

#### 2.3.3. Chromatographic Conditions for FEX

Assay of FEX was performed on a LiChrospher®CN column (125 × 4.0 mm, 5 *μ*m) purchased from Merck. The mobile phase consisted of acetonitrile, methanol and acetate buffer of pH 3.1 (30 : 30 : 40, v/v/v), sodium hexanesulfonate (5 mM), and TEA (0.1%). The flow rate of the mobile phase was 2.0 ml/min. Detection was done at 220 nm, while papaverine was used as the i.s.

#### 2.3.4. System Suitability

Six working solutions of TER were prepared by dispensing 0.6 ml volumes from the stock solution to 10 ml volumetric flasks to reach the concentration of 60 *μ*g/ml. To each flask, 0.8 ml of the i.s. solution was added.

Six working solutions of FEX were prepared by dispensing 1.0 ml from the stock solution to 10 ml volumetric flasks to reach the concentrations 100 *μ*g/ml. To each flask, 0.5 ml of the i.s. solution was added.

After adjusting with methanol to the mark, 6 injections from each working solution of TER or FEX were made onto the column.

#### 2.3.5. Specificity

Specificity of the methods was examined by determination of TER and FEX in the samples subjected to degradation under extreme conditions (1 M HCl and 1 M NaOH at 70°C for 180 min). It was confirmed as the ability to determine of no degraded TER or FEX in the presence of potential degradation products.

#### 2.3.6. Linearity for TER

Working solutions of TER were prepared by dispensing 0.05–0.6 ml of the stock solution to 10 ml volumetric flasks to reach the concentration range 5–60 *μ*g/ml. To each flask, 0.8 ml of the i.s. solution was added. After adjusting with methanol to the mark, five injections from each working solution were made onto the column. The ratios of peak areas of TER versus i.s. were plotted against the corresponding concentrations of TER.

#### 2.3.7. Linearity for FEX

Working solutions of FEX were prepared by dispensing 0.1–1.0 ml of the stock solution to 10 ml volumetric flasks to reach the concentration range of 10–100 *μ*g/ml. To each flask, 0.5 ml of the i.s. solution was added. After adjusting with methanol to the mark, five injections from each working solution were made onto the column. The ratios of peak areas of FEX versus i.s. were plotted against the corresponding concentrations of FEX.

#### 2.3.8. Accuracy for TER

Accuracy of the method was examined using the standard addition technique. Due to lack of commercial formulations containing TER, the model mixtures were prepared by mixing 60 mg of TER (50% addition), 120 mg of TER (100% addition), and 180 mg of TER (150% of addition) with 120 mg of starch. All mixtures were ground with a hand pestle for 30 min. The weighed portions containing 10 mg of TER were transferred to 10 ml volumetric flasks with ca. 8 ml of methanol, sonicated for 30 min, diluted to the mark, and filtered by nylon membrane filters (0.45 *μ*m). Then, 0.3 ml volumes of the filtrated solutions were mixed with 0.8 ml of the i.s. solution, diluted to 10 ml with methanol, and analyzed by the HPLC method described above. The assay was repeated three times at each level of addition, individually weighing the respective powdered mixture.

#### 2.3.9. Accuracy for FEX

Similarly, the model mixtures of FEX were prepared by mixing 60, 120, and 180 mg of the drug with 120 mg of starch to obtain 50, 100, and 150% of the tested concentration. The weighed portions containing 10 mg of FEX were transferred to 10 ml volumetric flasks with ca. 8 ml of methanol, sonicated for 30 min, diluted to the mark, and filtered by nylon membrane filters (0.45 *μ*m). Then, 0.5 ml volumes of the filtrated solutions were mixed with 0.5 ml of the i.s. solution, diluted to 10 ml with methanol, and analyzed by the HPLC method described above. The assay was repeated three times at each level of addition, individually weighing the respective powdered mixture.

#### 2.3.10. Precision of the Methods

The working solutions of TER were prepared by dispensing 0.15, 0.35, and 0.55 ml of the stock solution to 10 ml volumetric flasks to reach the concentrations 15, 35, and 55 *μ*g/ml. To each flask, 0.8 ml of the i.s. solution was added.

The working solutions of FEX were prepared by dispensing 0.15, 0.55, and 0.85 ml of the stock solution to 10 ml volumetric flasks to reach the concentrations 15, 55, and 85 *μ*g/ml. To each flask, 0.5 ml of i.s. solution was added.

After adjusting with methanol to the mark, the injections from each working solution were made onto the column three times during the same day, on three subsequent days. The concentrations of TER or FEX were calculated using respective calibration equations and expressed as RSD for intraday and interday precision.

#### 2.3.11. Sensitivity of the Methods

The limits of detection (LOD) and the limits of quantification (LOQ) for TER and FEX were determined from the SD of the intercept and the slope of respective regression lines at low concentrations, using 3.3 and 10 factors for LOD and LOQ, respectively.

#### 2.3.12. Robustness of the Method for TER

Robustness study was performed changing the flow rate of the mobile phase over the range 0.8–1.2 ml/min, the buffer content over the range 15–25%, and the detection wavelength over the range 218–222 nm. Additionally, pH of the buffer was changed in the range 4.3–5.3. Throughout all experiments, factors were changed one at a time, and finally, the differences in the peak shapes, peak areas, retention times, and resolution between TER and i.s. were estimated.

#### 2.3.13. Robustness of the Method for FEX

Robustness study was performed changing the flow rate of the mobile phase over the range 1.8–2.2 ml/min, the contents of the buffer over the range 35–45%, while the detection wavelength was changed in the range 218–222 nm. Additionally, the content of TEA in the mobile phase varied in the range 0.05–0.15%. Throughout all experiments, factors were changed one at a time, and finally, the differences in the peak shapes, peak areas, retention times, and resolution between FEX and i.s. were estimated.

### 2.4. Degradation and Analysis of the Stressed Samples

#### 2.4.1. Degradation at Different pH and High Temperature

From the stock solutions of TER and FEX, 1 ml was dispensed to small glass tubes (Medlab, Raszyn, Poland). To each tube, 1 ml of appropriate stressor (1 M HCl, 1 M NaOH, buffers of 4.0, 7.0, and 10.0) was added. The tubes were tightly closed with stoppers and placed in a thermostated water bath (WSL, Warszawa, Poland) set at 70°C. The samples were removed from the bath after subsequently 15, 30, 45, 60, 75, 90, 105, 120, 135, 150, 165, 180, 195, 210, 225, 240, 255, 270, 285, and 300 min. They were immediately cooled, neutralized if necessary, and diluted to 5 ml with methanol.

#### 2.4.2. Degradation at Different pH under UV/VIS Light

Equal volumes of 1 ml of the stock solutions of TER or FEX were dispensed to standardized quartz glass cuvettes. To each cuvette, 1 ml of appropriate stressor, i.e., buffers of 4.0, 7.0, and 10.0, was added. The cuvettes were tightly closed with stoppers and placed in a Suntest CPS Plus chamber (ATLAS, Linsengericht, Germany). The samples were exposed to UV/VIS light in the range 300–800 nm, with energy equal to 18902, 37804, 56706, 75608, and 94510 kJ/m^2^. These doses were attained during 7, 14, 21, 28, and 35 h of irradiation in the chamber. The energy of 18902 kJ/m^2^ was equivalent to 1.200.000 lux·h and 200 W/m^2^ that is recommended by the ICH Q1B guidelines as a dose of light that confirms drug photostability, while the next doses were 2–5 times higher [25]. During a whole experiment, temperature in the chamber did not exceed 35°C. After irradiation, the samples were diluted to 5 ml with methanol.

#### 2.4.3. LC-UV Measurements of the Stressed Samples

From the stressed samples, 1.25 ml was dispensed to 5 ml volumetric flasks, mixed with respective quantities of i.s., diluted to the mark with methanol and analyzed using LC-UV methods described above. The procedures were repeated three times for each sample, and the concentrations of no degraded TER or FEX were calculated using respective calibration equations. Finally, percentage levels of degradation of drugs were calculated taking into account their starting concentrations.

#### 2.4.4. Kinetics

The concentrations of drugs remaining after each time point of stressing were calculated using respective calibration equations. Then, the concentrations of no degraded drug or logarithms of the concentration of no degraded drugs were plotted against time of degradation to obtain the equations *y* = *ax* + *b* and the determination coefficients *R*^2^, and in consequence to determine the reaction order. Then, further kinetic parameters, i.e., degradation rate constant (*k*), degradation time of 10% substance (*t*_90_), and degradation time of 50% substance (*t*_50_) were calculated. The *t*_90_ and *t*_50_ values were calculated from the equations *t*_90_ = 0.105/*k* and *t*_50_ = 0.693/*k*, respectively.

## 3. Results and Discussion

### 3.1. Method Development and Optimization

Two simple isocratic LC-UV methods were developed for determination of TER or FEX with satisfactory retention times, peak shapes, and resolution between the mentioned drugs and respective internal standards. During the development study, HPLC columns with C18, C8, and CN stationary phases were tried. For TER determination, only a C8 column gave rewarding effects as far as reasonable retention times were concerned. In the case of FEX, a CN column was stated as optimal because of low tailing factors and reasonable retention times. For both drugs, mobile phases composed of acetonitrile, methanol, and acetate buffers occurred as sufficiently effective.

While the method for FEX was developed, addition of 0.05% TEA was effective for better resolution of the drug and i.s. as well as for reduction of the peak tailing. Also, an additive such as sodium hexanesulfonate had to be used to improve retention of FEX and obtain the *t*_R_ values above 2 min. Sodium hexanesulfonate is a low-molecular-weight alkylsulfonate that is used as an ion pairing reagent for HPLC and as an anionic surfactant. Its anionic sulfonate counterion permits the separation and resolution of positively charged analytes. Because of its low-molecular-weight, it does not form micelles in solutions. Nevertheless, it is worth mentioning that submicellar and micellar liquid chromatography (MLC) can be efficient alternative to conventional reversed-phase HPLC, with a great variety of interactions and, in consequence, major implications in retention and selectivity. The main strength of MLC is its capability of performing separation of mixtures containing cationic, anionic, and uncharged solutes, using isocratic elution [29]. Thus, many MLC methods were reported for determination of a wide range of compounds in different pharmaceutical preparations and pure drug substances. Additionally, many stability-indicating MLC methods were developed to study the degradation behavior of some pharmaceutical compounds [30]. It is clear that other analytical methods such as FT-IR and LC-MS could be used for other types of stability experiments, e.g., for identification of degradation products [31].

**Figure 2:**
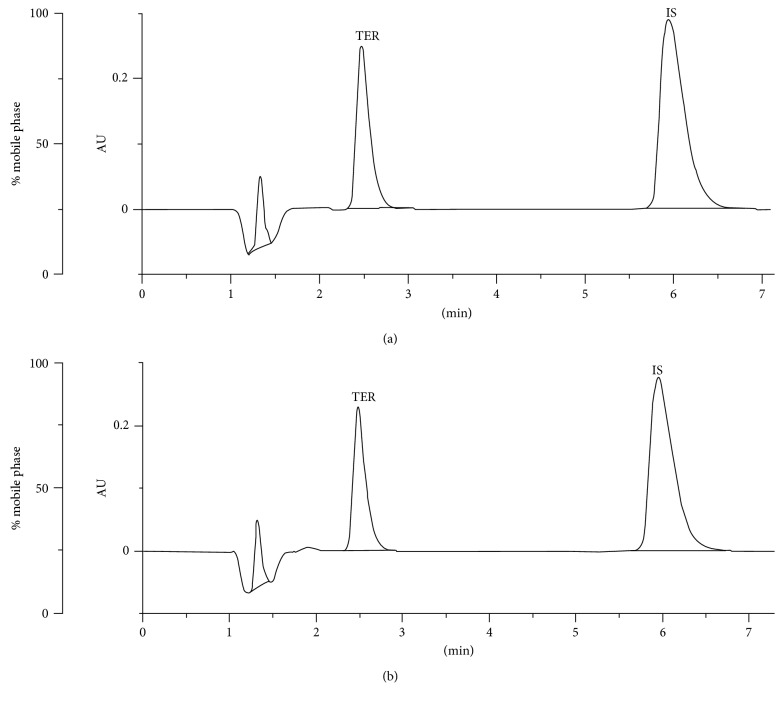
Typical chromatograms of terfenadine (TER) and internal standard (i.s.) in (a) the calibration solution and (b) the sample stressed in 1 M NaOH at 70°C for 180 min.

**Figure 3:**
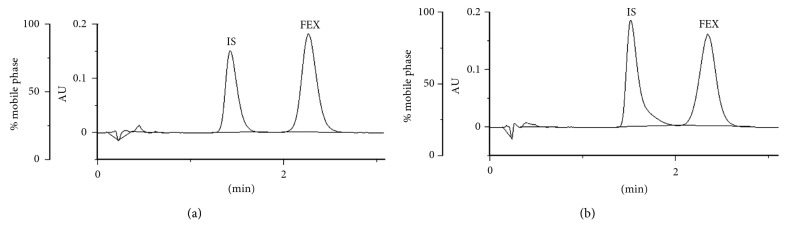
Typical chromatograms of fexofenadine (FEX) and internal standard (i.s.) in (a) the calibration solution and (b) the sample stressed in 1 M NaOH at 70°C for 180 min.

Finally, for six replicated injections of the both drugs, the average retention times were found to be 2.52 ± 0.02 min and 2.44 ± 0.03 min (±SD) for TER and FEX, respectively. The peaks were rather sharp and sufficiently separated from a baseline (Figures 2–3(a)).

#### 3.1.1. System Suitability

System suitability was established by 6 determinations of the solutions at the 100% concentrations on the same day, and the acceptance criteria were estimated as repeatability of peak areas and satisfactory tailing factors. The calculated RSD values for peaks areas were 0.87% and 0.61%, while the peak tailing values were 1.38 and 1.18 for TER and FEX, respectively (Table 1). Thus, the acceptance criteria as RSD below 1% and the peak tailing of not higher than 2 were fulfilled [28].

#### 3.1.2. Selectivity of the Methods

When the chromatograms obtained for the stressed samples of TER and FEX were examined, it was observed that there were no coeluting peaks at the retention time of TER or FEX, confirming selectivity of the method (Figures 2–3(b)).

#### 3.1.3. Linearity and Sensitivity for TER

The method for TER was found to be linear over the concentration range of 10–60 *μ*g/ml, with a linear equation *y* = 0.014252*x* − 0.004228 and average determination coefficient *R*^2^ of 0.9976. The LOD and LOQ, calculated from the standard deviation of the intercept and slope of the regression line, were 0.42 *μ*g/ml and 1.28 *μ*g/ml (Table 1).

#### 3.1.4. Linearity and Sensitivity for FEX

The method for FEX was found to be linear over the concentration range of 10–100 *μ*g/ml with a linear equation *y* = 0.010192*x* − 0.003933, with average determination coefficient *R*^2^ of 0.9996. The LOD and LOQ, calculated from the standard deviation of the intercept and slope of the regression line, were 1.35 *μ*g/ml and 4.09 *μ*g/ml (Table 1).

#### 3.1.5. Accuracy and Precision

The percentage recovery of added TER was calculated for each of the replicate sample. As a result, percentage recovery at three levels of addition in the range 98.21–101.96% was obtained. While precision of the method was examined at three concentrations, the RSD values in the range 1.01–1.78% (the one-day precision) and 1.04–1.97% (the interday precision) were obtained (Table 1).

As for FEX, % recovery at three levels of addition in the range 98.39–102.01% was obtained. While precision of the method was examined, the RSD values in the range 0.57–1.78% (the one-day precision) and 0.86–1.85% (the interday precision) were obtained (Table 1).

Thus, the results of percentage recoveries as well as RSD values for the both TER and FEX were within the acceptable limits from 98–102% and not more than 2.0%, respectively [27, 28].

#### 3.1.6. Robustness of the Methods

Robustness study for TER was performed changing the flow rate of the mobile phase over the range 0.8–1.2 ml/min, the buffer content over the range 15–25%, and the detection wavelength over the range 218–222 nm. Uniformity of the obtained peak areas, *t*_R_ values, and resolution between the peaks of interest confirmed the robustness of the method. However, when pH of the buffer was changed in the range 4.3–5.3, the obtained tailing factors for the i.s. indicated sensitivity of the method to small changes of pH.

Robustness study for FEX was performed changing the flow rate of the mobile phase over the range 1.8–2.2 ml/min, the buffer content over the range 35–45%, while the detection wavelength was changed in the range 218–222 nm. Uniformity of the obtained peak areas, *t*_R_ values, and resolution between the peaks of interest confirmed the robustness of the method. However, when the content of TEA in the mobile phase varied in the range 0.05–0.15%, the obtained *t*_R_ values and peak shapes for both FEX and i.s. were changed.

### 3.2. Comparative Study of Chemical Stability

Because of the limited use of TER in conventional therapy, there are not new reports concerning its analytical investigations. As a consequence, its chemical stability has not been studied so far. However, in the literature, there are reports showing new therapeutic recommendations for TER in view of its anticholinergic, antiviral, and anticancer activities [6–10]. In addition, there are still attempts to obtain new derivatives of TER and FEX with higher activities and lower side effects [3]. What is more, some reports indicating chemical instability of FEX has been published [15, 16, 19, 21–24]. Bearing in mind that all active pharmaceutical substances, and in consequence respective medicinal products, should maintain the appropriate potency and purity throughout their availability on the market, the need to obtain new active pharmaceutical substances with higher chemical stability is obvious. Therefore, we decided to perform a comparative study of chemical stability of TER and FEX, bearing in mind their chemical structures. Because some previous studies from the literature confirmed stability of FEX in a solid state [21, 22], all present experiments were performed in solutions. We were also guided by the premise that FEX, similar to many H_1_ antihistaminic drugs, can be administered in the form of medical solutions and suspensions.

Using new validated LC-UV methods, the concentrations of remaining (no degraded) TER and FEX were determined and percentage degradation of the drugs was calculated taking into account their starting concentrations. Because percentage degradation of both TER and FEX was higher than 10% during 300 min, kinetics of degradation was estimated for all stressed samples.

#### 3.2.1. Effects of pH and High Temperature

According to the literature, the highest degradation of FEX occurred in 0.1–1 M HCl and 0.1–1 M NaOH at room temperature (degradation above 95%) [16, 22]. On the contrary, degradation levels of 17.49% in 0.5 M HCl at 80°C and 10.66% in 0.5 M NaOH at 80°C were reported [21]. In the next paper, FEX was described as stable in acidic medium (0.29% of degradation) but sensitive to alkaline conditions (84.63% of degradation) [19]. Our study confirmed that FEX could degrade in a wide pH range from 1 to 14. The percentage levels of degradation varied from 40.42% (buffer of pH 7.0) to 72.98% (1 M NaOH). However, TER occurred much stabile than FEX in the same pH conditions because its degradation did not exceed 21.02% (1 M NaOH) (Table 2).

**Figure 4:**
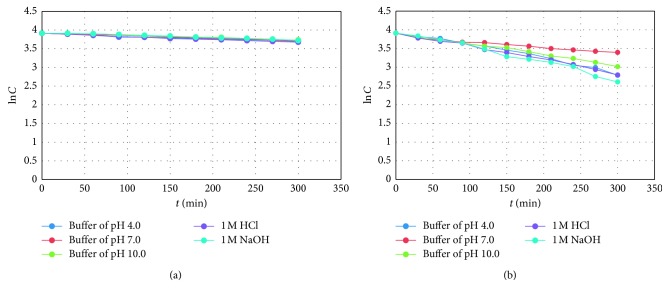
First-order plots of (a) terfenadine (TER) degradation at 70°C at different pH and (b) fexofenadine (FEX) degradation at 70°C at different pH.

Bearing in mind the highest degradation of FEX in strong alkaline medium, it can be supposed that its carboxylic group in its ionized form could increase susceptibility to degradation. In the literature, only one study concerning kinetic measurements for FEX degradation was found [23]. The degradation processes in 2 M HCl and 2 M NaOH at high temperature were described as the first-order reactions with *t*_50_ of 1.18 h in acidic and 2.82 h in alkaline medium. Our study showed strong correlations (high *R*^2^ values) for the plots of logarithms of concentration of no degraded FEX versus time of degradation, confirming the first-order kinetics. The calculated *t*_50_ values varied from 1.39 h (1 M HCl) to 1.17 h (1 M NaOH), confirming the lowest stability of FEX in alkaline medium. Degradation of TER also followed the first-order kinetics, i.e., high *R*^2^ values for the plots of logarithms of concentration of no degraded TER versus time of degradation (Figure 4).

For both drugs, the rate constants of degradation were at the levels of 10^−3^ min^−1^. However, TER showed the *t*_50_ values 3–6 times longer and the *t*_90_ values 2-3 times longer than FEX. The biggest differences were observed in 1 M HCl, buffer of pH 4.0, and 1 M NaOH.

We also observed that degradation rate constants of TER decreased when the pH increased from 1 to 14. At the same time, the degradation rate constants of FEX did not change significantly in the pH range 1–4 but increased when the pH was above 7.0. As a consequence, the shortest *t*_50_ value (1.17 h) was calculated in 1 M NaOH (Table 2).

TER has one ionizable group corresponding to the substituted piperidine ring, contributing to a pKa value of 8.85. FEX shows a zwitterionic structure with one carboxyl group (pKa = 4.25) and one piperidine ring (pKa = 9.35). Thus, it is expected that TER is isocationic in pH range less than 9 and neutral above 9 (Figure 1(b)), while FEX is isocationic in pH less than 4, neutral in the pH range from 4 to 9, and isoanionic in pH above 9 (Figure 1(c)). Thus, higher susceptibility to degradation for ionized forms of TER and FEX could be supposed. Especially, high susceptibility of FEX to alkaline degradation because of the presence of the carboxylic group was confirmed.

#### 3.2.2. Effects of pH and UV/VIS Light

In the literature, only scarce information is available concerning photostability of FEX, while no information about photostability of TER is present. In previous studies concerning FEX, natural sun light [16, 21, 22] or the light sources emitting UV light (254 nm) [15, 21] was used to examine stability of the drug in methanol or methanol-water solutions. No decomposition was seen after exposure of the drug to natural sun light for 8–46 h as well as for one week [16, 21]. Also, negligible degradation was observed after 8 h under UV light [21].

According to the next reports, irradiation in the region 350–650 nm was used to degrade FEX in the buffers of pH 6 and 11. During 6 h of experiment, around 70–80% of degradation was seen in both buffers. In addition, the kinetics of photodegradation was calculated, using the plots of concentrations, logarithms of concentration, and reciprocals of concentration of the remaining drug versus time of irradiation. It was stated that photodegradation of FEX could be described by the second-order kinetics with the rate constants at the level of 10^−5^ min^−1^ and the *t*_90_ values at the levels of 6.52–9.79 min [15, 24].

In our study, both TER and FEX were subjected to UV/VIS light in the region 300–800 nm in solutions covering the pH range 4–10 (Table 3).

**Figure 5:**
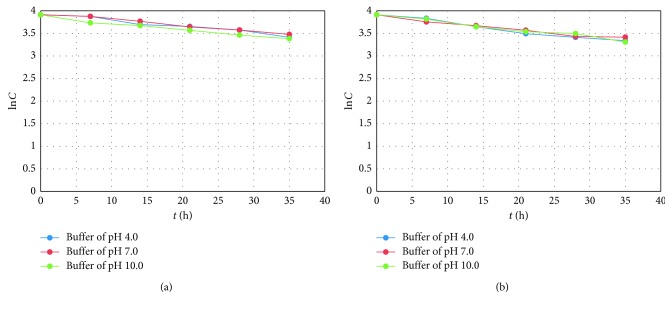
First-order plots of (a) terfenadine (TER) degradation under UV/VIS light at different pH and (b) fexofenadine (FEX) degradation under UV/VIS light at different pH.

The doses of light were equal or 2–5 times higher than a routine dose of light that confirms photostability of new drugs, according to the ICH Q1B guidelines [25]. Our study showed that strong correlations (high *R*^2^ values) were obtained for the plots of logarithms of concentration of no degraded drugs versus time of degradation, confirming that photodegradation of TER and FEX followed the first-order kinetics (Figure 5).

For the both drugs, the rate constants of photodegradation were higher and the calculated *t*_90_ values lower than previously reported [15, 24]. In addition, degradation of FEX was higher by approximately 4% than degradation of TER at all pH values checked (39.14–45.42% versus 35.32–41.06%) (Table 3). At the same time, it could be supposed that the presence of the carboxyl group in the structure of FEX was of less importance for stability under UV/VIS light than under high temperature at similar pH conditions.

## 4. Conclusions

As was described above, in the literature, there was not any report concerning chemical stability of TER. Thus, the results presented here supplemented the literary resources in this area. In addition, some new results concerning chemical stability of FEX were reported, and finally, higher stability of TER than FEX was shown. The presence of the carboxylic group in the structure of FEX seems to lower its affinity and toxicity to the cardiovascular system. On the contrary, FEX undergoes ionization in a wider pH range than TER and is much more sensitive to degradation.

Bearing in mind the persistent need for obtaining better drugs, the presented results could be helpful in developing new chemical derivatives with higher activity, lower side effects, as well as higher chemical stability. This information can be important when such new derivatives are projected not only in the field of their antihistaminic action, but also anticholinergic, antiviral, and anticancer activities.

## Figures and Tables

**Table 1:** Validation of LC-UV methods for determination of terfenadine (TER) and fexofenadine (FEX).

Parameter	TER	FEX
Retention time (min)	2.52	2.44
Internal standard (i.s.)	Amiodarone	Papaverine
Resolution (between the drug and the i.s.)	4.86	3.87
Linearity range (*μ*g/ml)	10–60	10–100
Slope	0.014252	0.010192
SD of slope	0.00016	0.000090
Intercept	−0.004228	−0.003933
SD for intercept	0.006410	0.002020
*R* ^2^	0.9976	0.9996
SD of *R*^2^	0.00066	0.00036
LOD (*μ*g/ml)	0.42	1.35
LOQ (*μ*g/ml)	1.28	4.09
Accuracy (% recovery)	98.21–101.96	98.39–102.01
Precision (RSD)		
Intraday	1.01–1.78	0.57–1.78
Interday	1.04–1.97	0.86–1.85
System suitability (RSD for peaks areas)	0.87	0.61
Tailing factor	1.38	1.18

**Table 2:** Percentage degradation and kinetic parameters of terfenadine (TER) and fexofenadine (FEX) at 70°C and different pH.

Stress conditions	Degradation (%)	Linear equation *y* = *ax* + *b*	*R* ^2^	k (min^−1^)	*t*_90_ (h)	*t*_50_ (h)
TER						
1 M HCl	21.02	*y* = −0.0008*x* + 3.9036	0.9893	1.84 × 10^−3^	0.95	6.27
pH 4.0	19.72	*y* = −0.0007*x* + 3.9015	0.9683	1.61 × 10^−3^	1.08	7.16
pH 7.0	18.44	*y* = −0.0007*x* + 3.9145	0.9948	1.61 × 10^−3^	1.08	7.16
pH 10.0	17.54	*y* = −0.0007*x* + 3.9300	0.9836	1.61 × 10^−3^	1.08	7.16
1 M NaOH	16.50	*y* = −0.0006*x* + 3.9258	0.9834	1.38 × 10^−3^	1.27	8.36
FEX						
1 M HCl	67.32	*y* = −0.0036*x* + 3.9210	0.9938	8.29 × 10^−3^	0.21	1.39
pH 4.0	67.54	*y* = −0.0037*x* + 3.9680	0.9846	8.52 × 10^−3^	0.21	1.36
pH 7.0	40.42	*y* = -0.0016*x* + 3.8485	0.9724	3.68 × 10^−3^	0.47	3.14
pH 10.0	59.14	*y* = −0.0028*x* + 3.9034	0.9922	6.45 × 10^−3^	0.27	1.79
1 M NaOH	72.98	*y* = −0.0043*x* + 3.9801	0.9839	9.90 × 10^−3^	0.18	1.17

**Table 3:** Percentage degradation and kinetic parameters of terfenadine (TER) and fexofenadine (FEX) under UV/VIS irradiation at different pH.

Stress conditions	Degradation (%)	Linear equation *y* = *ax* + *b*	*R* ^2^	k (min^−1^)	*t*_90_ (h)	*t*_50_ (h)
TER						
pH 4.0	39.32	*y* = −0.0142*x* + 3.9932	0.9656	5.45 × 10^−4^	3.21	21.19
pH 7.0	35.32	*y* = −0.0131*x* + 3.9365	0.9861	5.03 × 10^−4^	3.48	22.96
pH 10.0	41.06	*y* = −0.0145*x* + 3.8755	0.9811	5.56 × 10^−4^	3.14	20.77
FEX						
pH 4.0	43.64	*y* = −0.0175*x* + 3.9115	0.9756	6.72 × 10^−4^	2.60	17.19
pH 7.0	39.14	*y* = −0.0145*x* + 3.8787	0.9706	5.56 × 10^−4^	3.14	20.77
pH 10.0	45.42	*y* = −0.0167*x* + 3.9118	0.9795	6.41 × 10^−4^	2.73	18.02

## Data Availability

The data used to support the findings of this study are included within the article.
